# Social efficiency deficit deciphers social dilemmas

**DOI:** 10.1038/s41598-020-72971-y

**Published:** 2020-09-30

**Authors:** Md. Rajib Arefin, K. M. Ariful Kabir, Marko Jusup, Hiromu Ito, Jun Tanimoto

**Affiliations:** 1 Interdisciplinary Graduate School of Engineering Sciences, Kyushu University, Fukuoka, 816-8580 Japan; 2 Department of Mathematics, University of Dhaka, Dhaka, 1000 Bangladesh; 3 Department of Mathematics, Bangladesh University of Engineering and Technology, Dhaka, 1000 Bangladesh; 4 Tokyo Tech World Research Hub Initiative (WRHI), Institute of Innovative Research, Tokyo Institute of Technology, Tokyo, 152-8550 Japan; 5 Department of International Health, Institute of Tropical Medicine, Nagasaki University, Nagasaki, 852-8523 Japan; 6 Faculty of Engineering Sciences, Kyushu University, Fukuoka, 816-8580 Japan

**Keywords:** Evolutionary theory, Social evolution

## Abstract

What do corruption, resource overexploitation, climate inaction, vaccine hesitancy, traffic congestion, and even cancer metastasis have in common? All these socioeconomic and sociobiological phenomena are known as social dilemmas because they embody in one form or another a fundamental conflict between immediate self-interest and long-term collective interest. A shortcut to the resolution of social dilemmas has thus far been reserved solely for highly stylised cases reducible to dyadic games (e.g., the Prisoner’s Dilemma), whose nature and outcome coalesce in the concept of dilemma strength. We show that a social efficiency deficit, measuring an actor’s potential gain in utility or fitness by switching from an evolutionary equilibrium to a social optimum, generalises dilemma strength irrespective of the underlying social dilemma’s complexity. We progressively build from the simplicity of dyadic games for which the social efficiency deficit and dilemma strength are mathematical duals, to the complexity of carcinogenesis and a vaccination dilemma for which only the social efficiency deficit is numerically calculable. The results send a clear message to policymakers to enact measures that increase the social efficiency deficit until the strain between what is and what could be incentivises society to switch to a more desirable state.

## Introduction

Evolutionary game theory aims to understand the puzzle of cooperation in social and biological systems^1–3^. A central tenet of the theory is situations known as social dilemmas in which immediate self-interest is in conflict with long-term collective interests^4,5^. Attempts to resolve social dilemmas have led to five mechanisms of social viscosity: kin selection, group selection, direct reciprocity, indirect reciprocity, and network reciprocity^6^. The effect of social viscosity is to make interactions between actors “stickier” and thus transform the underlying dilemma from a defection-favouring to a cooperation-favouring one. In the special case of dyadic games in which interactions involve two actors who each choose whether to cooperate or defect, the dilemma’s nature and outcome are knowable *ex ante* using the concept of dilemma strength^7,8^. Intuitively, dilemma strength measures how lucrative defection is in the presence of a cooperator and how hazardous cooperation is in the presence of a defector.

Although dyadic games constitute a special case, their importance is hard to overstate; they have been invoked in contexts as diverse as political crises^9^, interfirm alliances^10^, legal problems^11^, cultural diversity^12^, and water resource management^13^. Each dyadic game is typically defined using four payoffs. Actors for mutual cooperation and defection respectively receive the reward payoff *R* and the punishment payoff *P*. If a cooperator is exploited by a defector, then the former receives a sucker’s payoff *S*, while the latter receives the temptation payoff *T*. The order of payoffs determines the nature of the dilemma. If, for example, the order is $T>R>P>S$, then the game in question is the infamous Prisoner’s Dilemma. It is rarely recognised that the evolutionary dynamics remain invariant if the game is rescaled in terms of dilemma strength parameters, $D'_\text {g}=\frac{T-R}{R-P}$ and $D'_\text {r}=\frac{P-S}{R-P}$^7,14^. Because four payoffs can be mapped into the same two parameter values in infinitely many ways, dilemma strength parameters define the whole classes of evolutionarily equivalent dyadic games. In line with the aforementioned intuitive meaning of dilemma strength, all game classes for which $D'_\text {g}>0$ and $D'_\text {r}>0$ are conducive to defection and thus represent a variant of the Prisoner’s Dilemma.

Dilemma strength determines the nature and the outcome of dyadic games irrespective of whether these games are enriched with any of the five social-viscosity mechanisms^7,8^. Most real-world dilemmas, however, violate the dyadic format and thus lack an obvious analogue to the concept of dilemma strength. This already holds for widely applicable Public Goods Games as a multiplayer generalisation of the Prisoner’s Dilemma^15^, and extends to closely related common-goods exploitation games^16^. It is furthermore possible to conceive (i) two-player games with additional action choices, e.g., reward^17^ and punishment^17,18^, (ii) multiplayer games with multiple actions^19,20^, and (iii) games embedded into a spatial structure^21–25^. Complexity ultimately escalates when social dilemmas are modelled after major societal concerns encompassing corruption^26,26^, vaccine hesitancy^27–29^, traffic congestion^30–32^, and countless others^33–35^. In view of such widespread use of social dilemmas, and a limited scope of dilemma strength as an *ex ante* predictor, a key question is whether it is possible to conceptually generalise dilemma strength to cover the full spectrum of social dilemmas, and thus uniformly guide policymaking for a better society.

**Figure 1:**
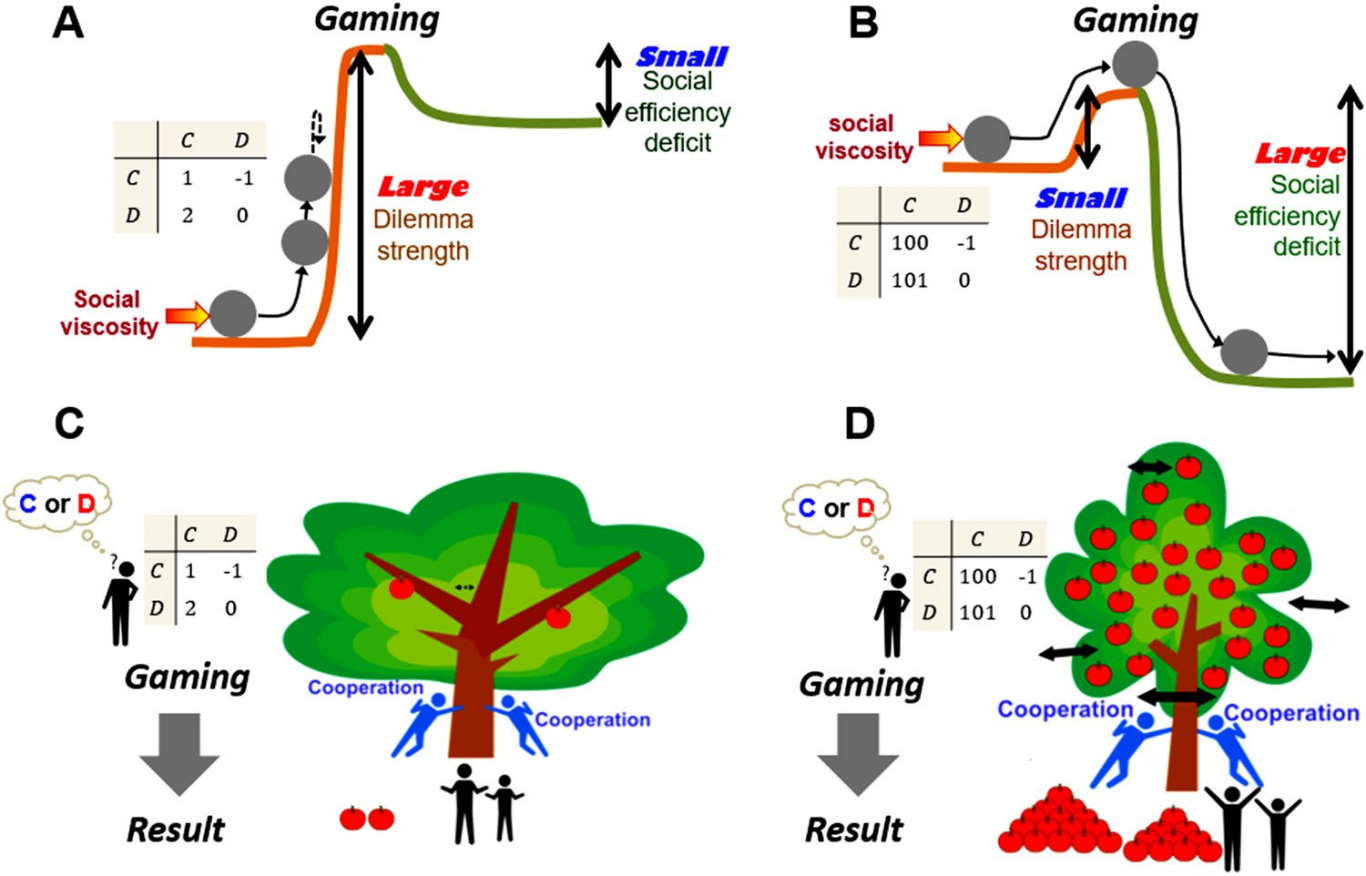
Social efficiency deficit generalises dilemma strength by quantifying the potential for the betterment of society. (**A**) Large dilemma strength firmly opposes the action of social-viscosity mechanisms, thus making it difficult for cooperation to evolve. The corresponding social efficiency deficit is small. (**B**) Small dilemma strength is readily counteracted by social-viscosity mechanisms, thus making it easy for cooperation to evolve. The corresponding social efficiency deficit is large. (**C**) When the social efficiency deficit is small, there is little room for the betterment through cooperation. Even if actors join forces, the tree grows only a few apples to pick. (**D**) When the social efficiency deficit is large, there is much room for the betterment through cooperation. Actors would be wise to join forces because the tree is brimming with apples to pick.

We show that a social efficiency deficit (abbreviated SED) generalises the concept of dilemma strength from dyadic games to social dilemmas of almost any complexity. The SED is defined as the difference between an actor’s average utility or fitness attained in (i) a social optimum and (ii) an evolutionary equilibrium. The social optimum maximises the average per-capita utility or fitness, whereas the evolutionary equilibrium emerges through evolutionary dynamics (see Methods for formal definitions). Accordingly, the larger the SED, the larger is the potential for the betterment of society when transitioning from the evolutionary equilibrium to the social optimum (Fig. 1). Such transitions in dyadic games, and especially the Prisoner’s Dilemma, are thought to be facilitated by one of the five mechanisms of social viscosity^6^. In more complex social dilemmas, e.g., the vaccination dilemma, it is policymakers or regulators who are intervening to induce transitions. We illustrate that interventions naturally split into an intrinsic and an extrinsic type, and that the SED is an easy-to-understand guide for policy planning regardless of whether a particular intervention is intrinsic or extrinsic.

## Results

### Social efficiency deficit in dyadic games

**Figure 2:**
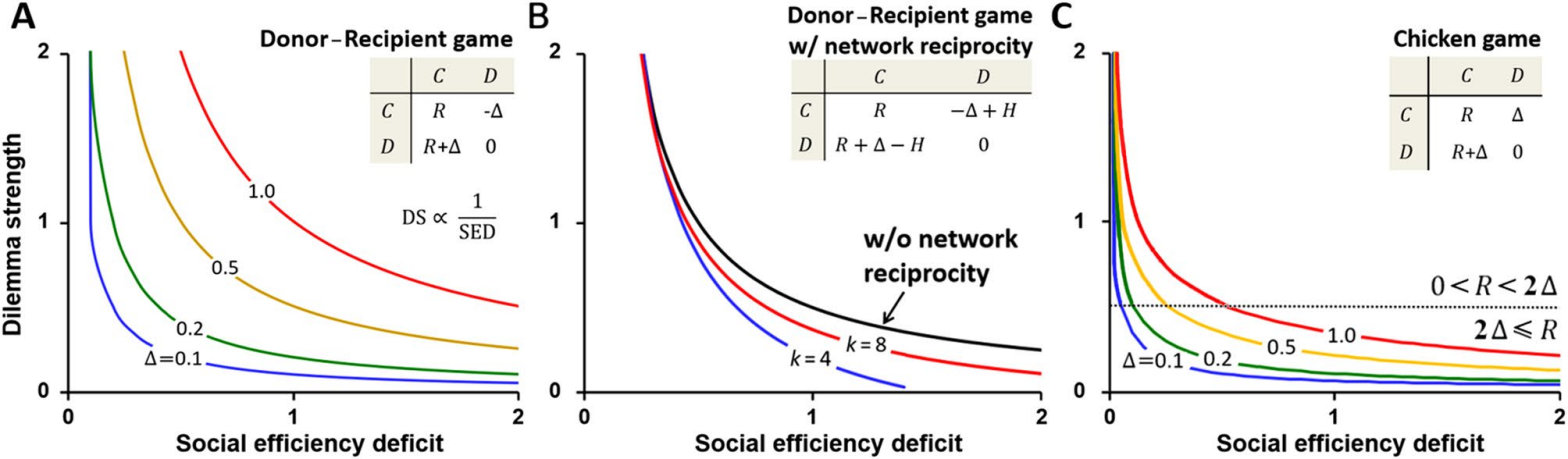
In dyadic games, dilemma strength and social efficiency deficit are the two sides of the same coin. (**A**) Shown is the payoff structure of the Donor-Recipient game, as well as the inverse relationship between dilemma strength and social efficiency deficit. As the latter increases, the former decreases, in which case even a weak incentive in the right direction may overturn defection. (**B**) Mechanisms of social viscosity may provide the incentive necessary to overturn defection. One such mechanism is network reciprocity, which modifies the payoff structure of the Donor-Recipient game by increasing a sucker’s payoff and decreasing the temptation payoff by an amount *H*. The quantity *H* increases with social efficiency deficit and decreases with the average number of neighbours in a network, *k*. Consequently, when *k* is sufficiently small, increasing social efficiency deficit may lower dilemma strength to zero, thus overturning defection and establishing cooperation. (**C**) Unlike all variants of the Prisoner’s Dilemma, including the Donor-Recipient game, which abhor the exploitation of cooperators by defectors, the Chicken game abhors mutual defection. Yet, the inverse relationship between dilemma strength and the social efficiency deficit remains qualitatively the same as before.

Dilemma strength predicts the outcome of dyadic games both in theory and practice. Introductory remarks already pointed out that because $D'_\text {g}$ and $D'_\text {r}$ respectively quantify how lucrative defection is in the presence of a cooperator and how hazardous cooperation is in the presence of a defector, all games satisfying $D'_\text {g}, D'_\text {r}>0$ are conducive to defection and are thus manifestations of the Prisoner’s Dilemma. Furthermore, when $D'_\text {g}>0$, but $D'_\text {r}\le {0}$, cooperating with a defector is no longer as hazardous, and we enter the domain of the Chicken game in which cooperators coexist with defectors. Conversely, when $D'_\text {g}\le {0}$, but $D'_\text {r}>0$, defecting against a cooperator is not as lucrative, and we enter the domain of the Stag Hunt game in which cooperators can prevail if their initial abundance is high enough. Finally, all games satisfying $D'_\text {g}, D'_\text {r}\le {0}$ are conducive to cooperation and are thus manifestations of the Harmony (i.e., Trivial) game. That dilemma strength is more than just a theoretical construct is evidenced by social-dilemma experiments. Human volunteers, for example, correctly perceive how strong a given dilemma is and act accordingly^36^. All this clearly emphasises the usefulness of dilemma strength in characterising dyadic games, but what about the SED? We hereafter establish a mathematical connection between the two concepts (Fig. 2) thereby demonstrating that the SED is as useful in the context of dyadic games as dilemma strength itself.

The Helping game^37^, also known as the Donor-Recipient game^38^, is a common representation of the Prisoner’s Dilemma that rose to prominence in the early work on indirect reciprocity^39^. The payoff structure is $R>0$ for mutual cooperation, $P=0$ for mutual defection, $S=-\Delta <0$ for cooperating with defectors, and $T=R+\Delta$ for defecting against cooperators; see Supplementary Information (SI) Appendix, Remark 1. From the aforementioned definitions of dilemma strength parameters, we have $D'_\text {g}=D'_\text {r}=\frac{\Delta }{R}$. At the same time, the social optimum is the state of full cooperation in which an actor’s average utility is *R*, whereas the evolutionary equilibrium corresponds to the Nash equilibrium of full defection in which an actor’s average utility is 0. The difference between these two utilities defines the SED, i.e., $\text {SED}=R$. We thus have that dilemma strength in the Donor-Recipient game is inversely proportional to the SED (Fig. 2A). Interpreting the SED as an opportunity cost that society incurs by settling for the evolutionary equilibrium instead of the social optimum, we see that defecting in the face of small dilemma strength is costly. Under such circumstances, even a weak incentive in the right direction may overturn defection into cooperation. Taking Fig. 1D as an example, the situation entails a substantial opportunity for cooperators. If actors fail to recognise the opportunity themselves, relatively little effort should suffice to persuade them to cooperate.

Overturning defection in dyadic games is often ascribed to one of the five mechanisms of social viscosity^6^. In the case of network reciprocity, for example, a dyadic game is enriched with social fabric in the form of a (complex) network that dictates who interacts with whom. Compared to the standard Donor-Recipient game, the same game played on the network has a larger sucker’s payoff, $S=-\Delta +H$, and a smaller temptation payoff, $T=R+\Delta -H$, where $H=H\left( R,k\right)$ increases with *R* and typically decreases with the average neighbourhood size in the network, *k*, commonly referred to as the average node degree^40^; see SI Appendix, Remark 2. A consequence of these modified payoffs is that dilemma strength parameters become $D'_\text {g}=D'_\text {r}=\frac{\Delta -H}{R}$, while the SED stays the same as before, i.e., $\text {SED}=R$. Zero dilemma strength may therefore be obtained for small enough average node degree by increasing the SED until $\Delta =H$ (Fig. 2B). At this point, cooperation replaces defection.

The Chicken game, also known as the Hawk-Dove game or the Snowdrift game, is another well-known dyadic game^41^, famously used as a metaphor for the nuclear stalemate^42^. In contrast to the Donor-Recipient game, a sucker’s payoff is positive here, $S=\Delta >0$, turning the payoff order into $T>R>S>P$. Being caught in mutual defection is thus worse than being exploited by a defector. Dilemma strength parameters are $D'_\text {g}=\frac{\Delta }{R}=-D'_\text {r}$, and evolution leads to a stable coexistence of cooperators and defectors; see SI Appendix, Remark 3. For the defined combination of payoffs, the equilibrium ratio of cooperators to defectors is 1:1, generating the average per-capita utility in the evolutionary equilibrium equal to $\frac{R+\Delta }{2}$. Meanwhile, the social optimum is twofold. If $0<R<2\Delta$, then a pair of cooperators obtains a lower collective payoff than a cooperator-defector pair. Full cooperation is socially suboptimal; instead there exists a frequency of cooperators $\frac{1}{2}<x_\text {m}<1$ that maximises the average per-capita utility at $\frac{1}{8\Delta }\left( R+2\Delta \right) ^2$. Accordingly, $\text {SED}=\frac{R^2}{8\Delta }$; see SI Appendix, Remark 3. Only when $2\Delta \le {R}$ does mutual cooperation become best for society, yielding a per-capita utility of *R*, and consequently $\text {SED}=\frac{R-\Delta }{2}$. For a fixed $\Delta$, dilemma strength thus once again decreases with an increasing SED (Fig. 2C), although quantitative details differ from before. The Chicken game is a particularly illustrative example because it reminds us that mutual cooperation may be socially suboptimal, and that by increasing the SED, policymakers try to destabilise the evolutionary equilibrium by widening the gap to the social optimum, whatever that optimum may be.

### Social efficiency deficit in Public Goods Games

Judiciary, sewerage, and parkland are but a few examples of public goods that permeate daily life. Their omnipresence, as well as their defining criteria of providing utility to everyone without being diminished by use, ensures that Public goods have a prominent spot in economic theory. The criterion of providing utility to everyone is called non-excludability, whereas the criterion of being undiminished by use is called non-subtractability^43^. The two criteria pose fundamental questions about who should contribute to the provision of public goods given that excluding non-contributors is difficult and, unlike with common goods, incentives for policing against overuse are non-existent. Attempts to answer such fundamental questions led to the formalisation of two key concepts, the voluntary contribution mechanism and free riding^44^. A series of empirical studies showed that free riding is not all-pervading despite increasing with repetition, and behaviours greatly differ between individual actors^45^.

**Figure 3:**
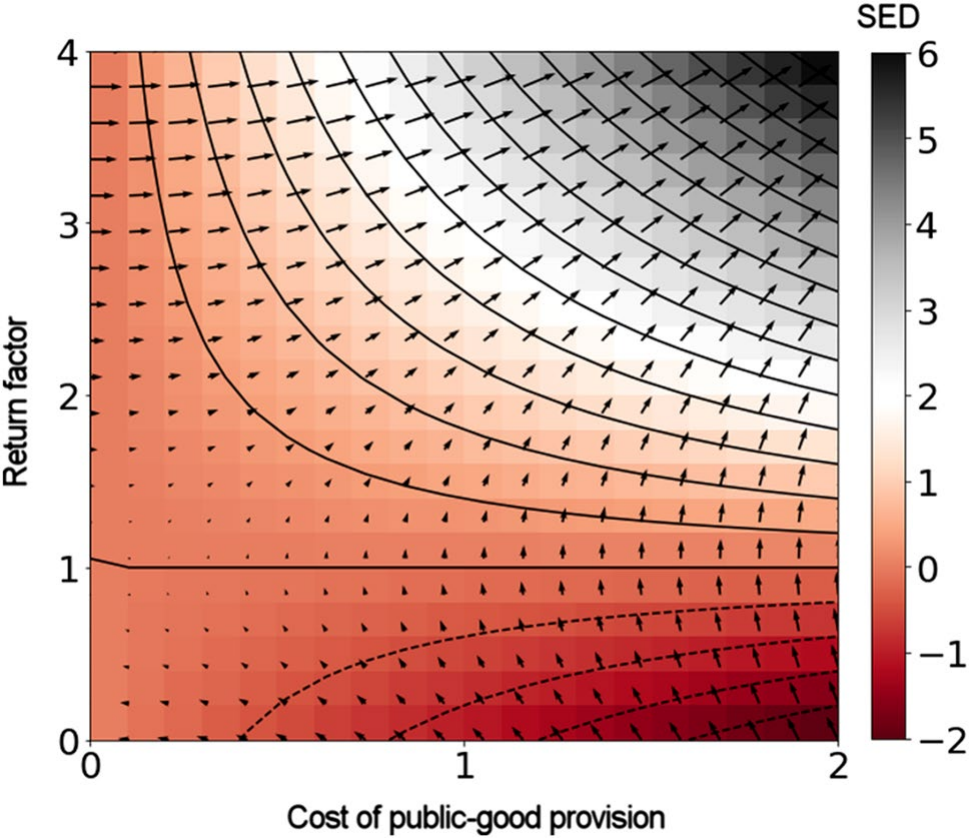
Social efficiency deficit informs policymaking. In Public Goods Games, the deficit depends on two game parameters, the cost of public-good provision $c'$ and the return factor *r*. Increasing both of these parameters aids overturning defection in favour of cooperation by strengthening the strain between the evolutionary equilibrium and the social optimum. This gives policymakers a general idea of a direction in which an intervention, if feasible, should push the value of parameters *r* and $c'$. The gradient map further indicates the direction along which an incremental intervention generates the biggest bang for the buck. In this context, the map reveals that public goods with return factors lower than unity are socially counterproductive, and policymakers should decrease $c'$ until securing $r>1$.

The voluntary contribution mechanism comprises a number of actors who, instead of relying on a central authority, themselves decide how much to provision to public goods. The cost of public-good provision, $c'$, is often assumed fixed so that decisions boil down to whether to contribute or not. The total provision grows by a return factor, *r*, before being divided equally among all actors irrespective of who contributed. The described setup is equivalent to a multiplayer generalization of the Donor-Recipient game; see SI Appendix, Remark 4. Accordingly, the Nash equilibrium is that of full defection, yielding an average utility equal to zero. Conversely, the social optimum is that of full cooperation, yielding the average utility equal to $\left( r-1\right) c'$. We thus have $\text {SED}=\left( r-1\right) c'$, which shows that the SED increases with both *r* and $c'$ (Fig. 3). Policymakers should aim to increase the SED with their policies in order to strengthen the strain between the evolutionary equilibrium of full defection and the social optimum of full cooperation. In this context, we define an intrinsic intervention as the one that affects the game parameters themselves, here, *r* and $c'$. Policymakers would thus want to ramp up *r* and $c'$ in a quantum-leap manner, i.e., as much and as quickly as possible. In realistic social-dilemma situations, however, inducing a quantum leap in parameter values may be unfeasible (legally, technologically, etc.). An incremental intervention may be called for, in which case a map of the SED gradient guides policymakers by showing the direction of the steepest increase in the SED along which policies generate the biggest bang for the buck (Fig. 3).

Diving deeper into the intricacies of policymaking, we can conclude that no amount of incremental intrinsic interventions is able to overturn defection in Donor-Recipient and Chicken games as defined herein. Increasing the SED can only prime the system for a change, but ultimately an extrinsic intervention in the form of a new game rule, e.g., repeated interactions or an image score^39^, is necessary to reach the desired state. The same, however, is not the case in the Donor-Recipient game with network reciprocity nor in Public Goods Games. In the latter, for instance, increasing the return factor *r* to a value that exceeds the number of participating actors, guarantees convergence to the social optimum of full cooperation; see SI Appendix, Remark 4. This reminds us that attaining an ever larger SED is not an objective *per se*, but rather a precursor of social change. It is furthermore illustrative to ask why the gradient of the SED suggests increasing both the return factor *r* and the cost of public-good provision $c'$ (top right corner in Fig. 3), when only the former guarantees convergence to the social optimum. The parameter $c'$, although immaterial to where the system converges, plays a role by setting the convergence rate. In the real world, policies that converge to the desired state at Platonic rates are unfeasible^26^.

The concept of public goods transcends the socio-economic domain. In the microscopic world, for example, microbial^46^ and tissue^47^ cell-cell interactions are mediated by public goods called diffusible factors. Cellular cooperation resulting from such interactions is instrumental to a range of human hazards, including antibiotic resistance^48^ and carcinogenesis^49^. For cells to cooperate, it is necessary to resolve the same fundamental conflict between selfish and collective interests that is embodied in all social dilemmas. This implies that the SED remains an equally useful concept regardless of whether the focus is on cells or humans, which we demonstrate by analysing the effects of diffusible factors on cancer-cell heterogeneity; see SI Appendix, Remark 5. The SED readily reveals that the insidious nature of carcinogenesis leaves only a narrow pathway to decisive therapies (Fig. S1).

### Complexity of a vaccination dilemma

The Chicken game has already demonstrated that a social optimum need not be fully cooperative. Next, we witness that even a fully cooperative social optimum need not be desirable in a complex world. One incarnation of such complexity is the vaccination dilemma, in which actors make a decision whether to vaccinate or not based on how they and their neighbours fared during past epidemics^28,29^; see SI Appendix, Remark 6. In short, contracting an infectious disease is burdensome and entails a cost of infection. Vaccines confer immunity from the disease, but with two caveats. First, getting vaccinated is costly, which is usually expressed in terms relative to the cost of infection, i.e., a vaccine costs $0\le c_\text {r}<1$. Second, even after getting vaccinated, immunity is all but guaranteed and depends on the vaccine’s efficacy $0<e\le 1$. According to these rules, when a generation of actors—some of whom are vaccinated—experiences an epidemic, there are four possible outcomes: (i) non-vaccinated and non-infected actors pay nothing, (ii) vaccinated and non-infected actors pay $c_\text {r}$, (iii) non-vaccinated and infected actors pay 1, and (iv) vaccinated and infected actors pay $c_\text {r}+1$. When the epidemic finally subsides, each actor compares their payoff with that of the neighbouring actors and decides how to act in the next generation. The decision may be to stick to the previous action or to imitate one of the neighbours, where the probability of imitation increases with the payoff difference between the actor and the neighbour.

**Figure 4:**
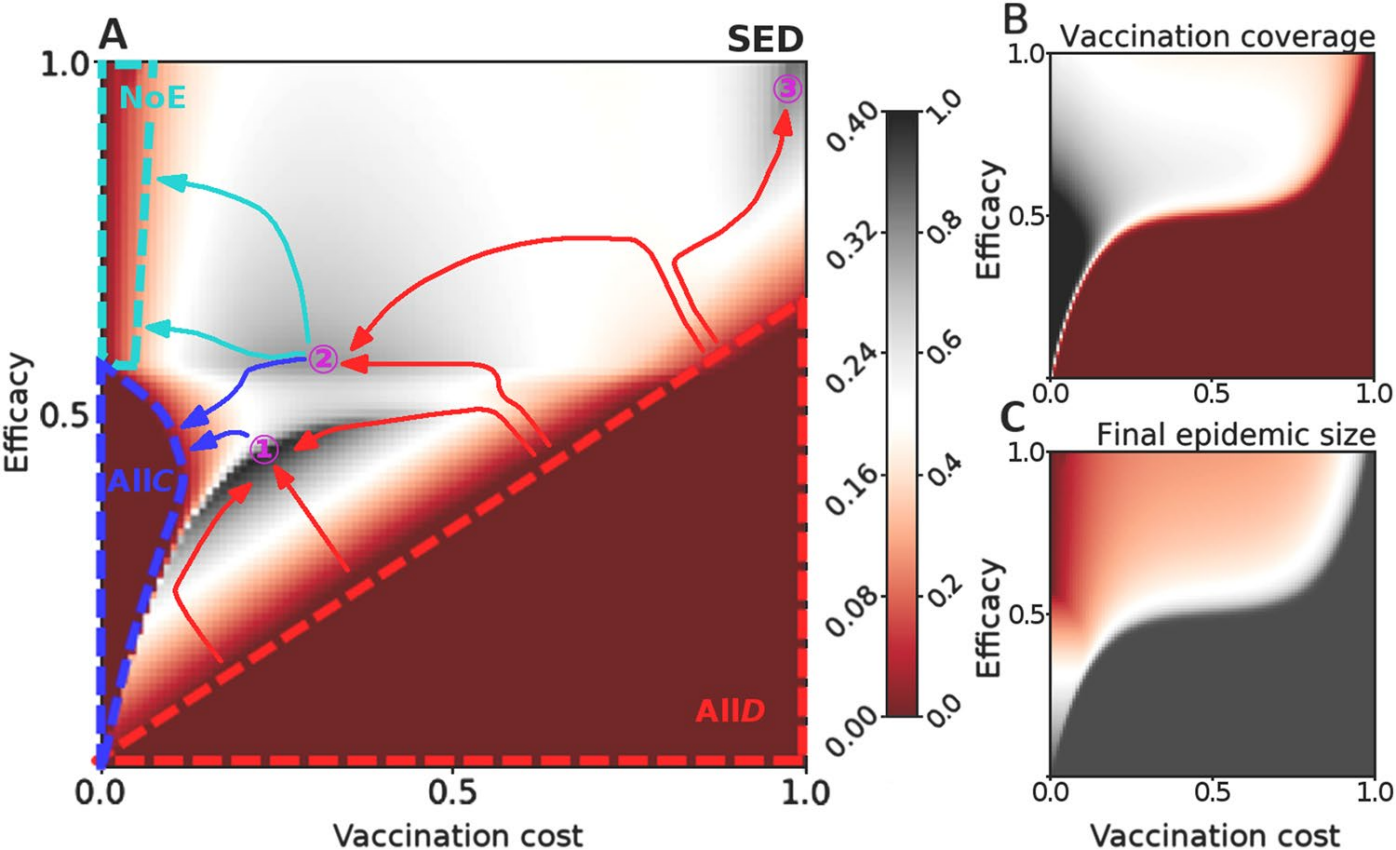
Social efficiency deficit reveals the complexity of the vaccination dilemma. (**A**) Depending on vaccine efficacy and the relative vaccination cost, there are three regions in which the evolutionary equilibrium and the social optimum coincide ($\text {SED}{=}0$). All*D* is fully defecting; nobody vaccinates because the vaccine is either too inefficacious or too expensive, or a combination thereof. All*C* is fully cooperative; everybody vaccinates because the vaccine is cheap, despite offering limited protection. NoE is partly cooperative; a fraction of the population vaccinates, but the vaccine is so cheap and efficacious that the epidemic is averted. The curved arrows represent incremental policies that are attempting to escape from All*D* (red) and approach All*C* (blue) or NoE (turquoise). (**B**) Vaccination coverage as a function of vaccine efficacy and the relative vaccination cost. A fully defecting region is dark red, whereas a fully cooperative region is black. (**C**) Final epidemic size as a function of vaccine efficacy and the relative vaccination cost. The epidemic is averted in the dark red region.

The complexity of the described setup is quickly revealed by the fact that there are three outcomes for which $\text {SED}=0$ (Fig. 4A). Because $\text {SED}=0$ signifies that the evolutionary equilibrium and the social optimum coincide, each of these three outcomes eliminates the dilemma. And yet, they could not be more different. The first of the three outcomes, denoted All*D* (Fig. 4A), is characterised by complete defection (i.e., nobody vaccinates; Fig. 4B) due to a vaccine that is either inefficacious or overly expensive, or a combination thereof. The second outcome, denoted All*C* (Fig. 4A), is fully cooperative (Fig. 4B) because the vaccine is so cheap that vaccinating makes sense despite the relatively low efficacy. Finally, the third outcome, denoted NoE (Fig. 4A), is only partially cooperative (Fig. 4B), but this is because the vaccine is so cheap and efficacious that even limited vaccination is sufficient to eliminate an outbreak (Fig. 4C). In terms of societal desirability, All*D* is disastrous, All*C* is acceptable, and NoE is ideal. We thus see that a fully cooperative social optimum need not be particularly attractive to policymakers.

Further policymaking considerations suggest that, as in previous examples, policies should aim to increase the SED in order to escape the undesirable All*D* state. Initially, increasing the SED involves reducing the vaccine cost and improving the vaccine efficacy, which is in line with intuition (red paths in Fig. 4A). After a while, however, the complexity of the real world kicks in, and policies make turns away from the intuitive direction. For example, when both cost and efficacy are low to moderate, increasing the SED ends in a maximum (point ① in Fig. 4A) in which actors underuse a vaccine that is just slightly too expensive for its efficacy. From here, it takes only a minor cost reduction to transition to the All*C* state and reach a sweet spot where the final epidemic size is substantially decreased (blue paths in Fig. 4A; cf. Fig. 4C). This sweet spot may be missed if the All*C* state is approached while the vaccine efficacy is too low, which is the reason why the leftmost red path in Fig. 4A steers away from the All*C* domain and towards point ①, thus suggesting that it is worthwhile incurring more cost to improve efficacy. Other circumstances, by contrast, suggest sacrificing some efficacy to reduce costs, which may again lead to the described point ①, but also to a situation in which actors now overuse a vaccine that is slightly too expensive for its efficacy (point ② in Fig. 4A). From here, it also takes only a minor cost reduction to transition either to the All*C* state or even to the most desirable NoE state (blue and turquoise paths in Fig. 4A). If reducing the vaccine cost, which is by definition an intrinsic intervention, is technologically unfeasible, policymakers can alternatively resort to an extrinsic intervention in the form of a subsidy. This reveals a surprising fact that society may want to subsidise what already constitutes a relatively inexpensive vaccine. Curiously, for vaccines that are expensive-yet-efficacious to begin with, increasing the SED suggests that the best policy is to ramp up efficacy no matter the cost (point ③ in Fig. 4A). Then even a small subsidy may recruit enough vaccinators to substantially reduce the final epidemic size (cf. Fig. 4C) until a cost-saving technological breakthrough presents itself in the future.

## Discussion

We have introduced a novel concept, termed social efficiency deficit (SED), that generalizes the idea of dilemma strength beyond dyadic games in order to decipher social dilemmas of any complexity. The SED is defined as a difference between an actor’s expected utility or fitness in (i) a social optimum vs. (ii) an evolutionary equilibrium. This definition suggests that the SED is an opportunity cost incurred when society evolves to a suboptimal equilibrium. If the SED is small (resp., large), then society stands to gain a little (resp., a lot) by overturning the evolutionary equilibrium in favor of the social optimum. It is important to recognize that social optima, even fully cooperative ones, need not be socially desirable. This is because social optima correspond to those behaviors that, for a given set of social-dilemma parameters, maximize the expected utility or fitness, while policymaking priorities may lie elsewhere. Aligning behavioral incentives in a socio-economic system with policymaking priorities involves, at least initially, increasing the SED to make the strain between what is (i.e., the current evolutionary equilibrium) and what could be (i.e., the desired social optimum) more apparent. This alignment is achieved by intervening to either change the intrinsic social-dilemma parameters, e.g., the payoffs of a Prisoner’s Dilemma, or to introduce an external rule, e.g., a Prisoner’s Dilemma with peer punishment^50^ or onymous encounters^51^.

Developing intuition about the discussed ideas is perhaps best served by briefly recapitulating a concrete example. In the vaccination dilemma, it is socially optimal to abstain from vaccination when a vaccine is inefficacious, overly expensive, or a combination thereof. This behavior, however, has a negative side-effect of maximizing the epidemic size, which clearly goes against the desire to protect public health. Policymakers who feel compelled to escape from such an undesirable social optimum have two options. When legally, technologically, or otherwise feasible, one option is intrinsic interventions that tune the social-dilemma parameters, i.e., vaccine efficacy or vaccination cost, to skew incentives in favor of an evolutionary equilibrium that is closer to the desired state. When intrinsic interventions are unfeasible, the alternative is to impose extrinsic rules, e.g., in the form of a subsidy. The key is to avoid naive interventions, be they intrinsic or extrinsic, that would induce a transition to another social optimum in which improvements to the situation are merely cosmetic. In the vaccination dilemma, for instance, even if everybody vaccinated with a cheap-yet-inefficacious vaccine, improvements to public health would be minimal. The SED avoids this type of decision-making pitfalls, oftentimes by identifying surprising tradeoffs such as purposefully sacrificing the vaccine cost for efficacy. In view of all this, it is safe to conclude that one of the main strengths of the SED is the ability to map naive interventions, navigate around them, and ultimately steer policymaking towards the true betterment of society.

## Methods

Here, we present a general definition of the social efficiency deficit (SED) that is also a step-by-step recipe for obtaining the SED in practice. Let us suppose that $S=\{s_1,...,s_n\}$ is a set of strategic decisions and that ${\mathbf {x}}=(x_1,...,x_n)^T\in \left[ 0,1 \right] ^n$ is the matching column vector of strategy abundances in a population of actors. Because abundances must sum to unity, we have a constraint $\Vert {\mathbf {x}}\Vert _1=\sum _{i=1}^n{x_i}=1$. Associated with the strategy-abundance vector ${\mathbf {x}}$ is the expected utility

1 $$\begin{aligned} {\overline{\pi }}\left( {\mathbf {x}} \right) =\sum _{i=1}^n{x_i\pi _i\left( {\mathbf {x}} \right) }=\sum _{i=1}^n{\sum _{j=1}^n{x_i x_j m_{ij}}}, \end{aligned}$$

where $\pi _i\left( {\mathbf {x}} \right) =\sum _{j=1}^n{x_j m_{ij}}$ is the expected utility of an actor choosing the strategy $s_i$, and $m_{ij}$ are the elements of a bilateral payoff matrix ${\mathbf {M}}=\left[ m_{ij} \right]$ that stipulates what happens when the actor using the strategy $s_i$ meets another actor using the strategy $s_j$. The social optimum is then defined as the vector of strategy abundances that maximizes the expected utility in Eq. (1)

2 $$\begin{aligned} \Pi _\text {SO}=\max _{ \begin{aligned}&{\mathbf {x}}\in \left[ 0,1 \right] ^n\\ \Vert&{\mathbf {x}}\Vert _1=1 \end{aligned} }{{\overline{\pi }}\left( {\mathbf {x}} \right). } \end{aligned}$$

To define the SED, we further need the expected utility in an evolutionary equilibrium. The evolutionary dynamics of abundances is given by differential equations of the form

3 $$\begin{aligned} \frac{\text {d}x_i}{\text {d}t}=F_i\left[ {\mathbf {x}}, \pi _i\left( {\mathbf {x}} \right) , {\overline{\pi }}\left( {\mathbf {x}} \right) ; \,t \right] , \end{aligned}$$

where $F_i=F_i\left[ {\mathbf {x}}, \pi _i\left( {\mathbf {x}} \right) , {\overline{\pi }}\left( {\mathbf {x}} \right) ; \,t \right]$ is a function of strategy abundances, expected utilities, and possibly time. In the case of the replicator dynamics, we have $F_i=x_i\left[ \pi _i\left( {\mathbf {x}} \right) -{\overline{\pi }}\left( {\mathbf {x}} \right) \right]$, but other, e.g., mean-field, dynamic equations are possible too. Upon identifying the stable equilibrium points ${\mathbf {x}}^*$, such that $F_i\left[ {\mathbf {x}}^*, \pi _i\left( {\mathbf {x}}^* \right) , {\overline{\pi }}\left( {\mathbf {x}}^* \right) ; \,t \right] =0$, the corresponding expected utility becomes $\Pi _\text {NE}={\overline{\pi }}\left( {\mathbf {x}}^* \right)$. If the equilibrium is multimorphic, we then perform additional averaging over all possible initial conditions ${\mathbf {x}}_0$

4 $$\begin{aligned} \Pi _\text {NE}=\int _{ \begin{aligned} \left[ 0,1 \right] ^n&\\ \Vert {\mathbf {x}}_0\Vert _1=&1 \end{aligned} }{{\overline{\pi }}\left[ {\mathbf {x}}^*\left( {\mathbf {x}}_0 \right) \right] \text {d}{\mathbf {x}}_0}. \end{aligned}$$

Finally, the SED is defined as a difference between expected utilities in the social optimum and the evolutionary equilibrium, i.e., the difference between expected utilities in Eqs. (2) and  (4)

5 $$\begin{aligned} \text {SED}\equiv \Pi _\text {SO}-\Pi _\text {NE}. \end{aligned}$$

## Supplementary information

Supplementary Information.
