# Supplementary material for: Social efficiency deficit deciphers social dilemmas
Source: Sci Rep. 2020 Sep 30;10:16092. doi: 10.1038/s41598-020-72971-y (PMC7527514; doi:10.1038/s41598-020-72971-y)
Supplement: Supplementary file 1 — Supplementary Information. [file 41598_2020_72971_MOESM1_ESM.pdf]

Supplementary Information for

Social efficiency deficit deciphers social dilemmas

Md. Rajib Arefin, K. M. Ariful Kabir, Marko Jusup, Hiromu Ito, Jun Tanimoto

Corresponding Authors: Md. Rajib Arefin, Marko Jusup, and Jun Tanimoto.
E-mails: arefin.math@du.ac.bd (Md. R. A.); mjusup@gmail.com (M. J.); tanimoto@cm.kyushu-u.ac.jp (J. T.)

This PDF file includes:

Supplementary text
Fig. S1
References for SI reference citations

Supporting Information Text

Supplementary Remark 1

Here, we provide additional information on the payoffs of the Donor-Recipient game. We start from a general bilateral payoff matrix for dyadic games

$$\mathbf{M}_{2\times 2} = \begin{pmatrix} R & S \\ T & P \end{pmatrix}, \qquad [1]$$

which signifies that a cooperator meeting another cooperator earns a payoff R, a cooperator meeting a defector earns a payoff S, a defector meeting a cooperator earns a payoff T, and a defector meeting another defector earns a payoff P. The Donor-Recipient game interprets the cooperator as a donor who incurs a cost $c > 0$ in order for the other actor, i.e., a recipient, to earn a benefit $b > c$. The defector in the Donor-Recipient game does nothing, and thus incurs zero cost. Based on these definitions, the following holds: $R = b - c$, $S = -c$, $T = b$, and $P = 0$. The payoff order is thus $T > R > P > S$, indicating that the Donor-Recipient game is indeed an instance of the Prisoner's Dilemma. To obtain the payoff matrix shown in Fig. 2A of the main text, we define $c = \Delta$, while keeping R as the reference payoff. Consequently, the bilateral payoff matrix of the Donor-Recipient game turns to

$$\mathbf{M}_{\text{D-R}} = \begin{pmatrix} R & -\Delta \\ R+\Delta & 0 \end{pmatrix}. \qquad [2]$$

The dilemma strength parameters follow straight from their definitions

$$D'_{\mathrm{g}} \equiv \frac{T-R}{R-P} = \frac{R+\Delta-R}{R} = \frac{\Delta}{R}, \qquad [3a]$$

$$D'_{\mathrm{r}} \equiv \frac{P-S}{R-P} = \frac{0+\Delta}{R} = \frac{\Delta}{R}. \qquad [3b]$$

To calculate the social efficiency deficit (abbreviated SED), we recognize that the social optimum is fully cooperative, in which case the expected per-capita utility is $\Pi_{\mathrm{SO}} = R$. The evolutionary equilibrium, by contrast, is fully defecting, meaning that the expected per-capita utility is $\Pi_{\mathrm{NE}} = 0$. The SED is the difference between these two utilities, thus becoming

$$\mathrm{SED} = \Pi_{\mathrm{SO}} - \Pi_{\mathrm{NE}} = R - 0 = R. \qquad [4]$$

Finally, defining $\mathrm{DS} \equiv D'_{\mathrm{g}} = D'_{\mathrm{r}}$, we see that $\mathrm{DS} \propto \mathrm{SED}^{-1}$, i.e., dilemma strength is inversely proportional to the SED.

Supplementary Remark 2

How is the general bilateral payoff matrix for dyadic games, $\mathbf{M}_{2\times 2}$, transformed into a bilateral payoff matrix, $\mathbf{M}_{\mathrm{NR}}$, for dyadic games on networks? In a well-mixed population every actor can meet every other actor, which in a network representation would correspond to a fully connected graph. If, however, it is imagined that some actors can never meet, then the corresponding connections must be removed from the graph, thus creating a social network. Instead of being connected to everyone, actors in the network maintain connections only with their neighbors. The average number of neighbors per actor, k, is called the average node degree of the network. The mere presence of a network is oftentimes sufficient to overthrow defection in favor of cooperation without the need for any additional strategic complexity. This effect is called network reciprocity (1). Exploring network reciprocity by analytical means is made possible by a number of simplifying assumptions (2):

- A pair-approximation technique is used to keep track of the local frequencies of cooperators and defectors. Local frequencies arise naturally in relation to the concept of neighborhood because a particular neighborhood need not faithfully reflect the global state of the system given by the global frequencies of cooperators and defectors. The key is to track only the local frequencies of actor pairs, i.e., of cooperators who have a cooperator in the neighborhood, cooperators who have a defector in the neighborhood, defectors who have a cooperator in the neighborhood, and defectors who have a defector in the neighborhood. What two-step neighbors do is irrelevant.
- By assuming weak selection, meaning that only a small fraction of payoff earned in the game is turned into utility or fitness, the dynamics of local frequencies becomes decoupled from the dynamics of global frequencies. The former always equilibrate before affecting the latter.
- Selecting a particular updating rule is necessary to specify how actors make their strategic decisions. An example of this is the death-birth rule, which recognizes two distinct events. First, a randomly selected actor in the network is assumed to die. The vacancy is then occupied by the offspring of a neighboring actor who is

randomly selected proportional to their fitness. The offspring of a cooperator (resp., defector) is a cooperator (resp., defector).

Under the above-listed assumptions, the off-diagonal elements of the general bilateral payoff matrix $\mathbf{M}_{2\times 2}$ get modified by a quantity (2)

$$H = \frac{(k+1)(R-P) - T + S}{(k+1)(k-2)}, \qquad [5]$$

which in the case of the Donor-Recipient game defined by Eq. [2] turns into

$$H = \frac{Rk - 2\Delta}{(k+1)(k-2)}, \qquad [6]$$

which, for a fixed $\Delta>0$, increases with R and decreases with $k > 2$. The payoff matrix is

$$\mathbf{M}_{\mathrm{NR}} = \begin{pmatrix} R & -\Delta + H \\ R + \Delta - H & 0 \end{pmatrix}. \qquad [7]$$

Following the definition of the dilemma strength parameters

$$D'_{\mathrm{g}} \equiv \frac{T-R}{R-P} = \frac{R + \Delta - H - R}{R} = \frac{\Delta - H}{R}, \qquad [8a]$$

$$D'_{\mathrm{r}} \equiv \frac{P-S}{R-P} = \frac{0 + \Delta - H}{R} = \frac{\Delta - H}{R}. \qquad [8b]$$

As long as $D'_{\mathrm{g}} = D'_{\mathrm{r}} > 0$, the game here is also an instance of the Prisoner's dilemma, implying that exactly the same argument as the one used to derive Eq. [4] still holds. Thus, we again have

$$\mathrm{SED} = \Pi_{\mathrm{SO}} - \Pi_{\mathrm{NE}} = R - 0 = R. \qquad [9]$$

We see from Eq. [6] that for a fixed Δ, there may be $k > 2$ such that $\mathrm{SED} = R \geq \Delta(k-1)$, yielding $H \geq \Delta$. At this moment $D'_{\mathrm{g}} = D'_{\mathrm{r}} \leq 0$ and the game exits the Prisoner's Dilemma domain.

Supplementary Remark 3

Unlike the Prisoner's Dilemma, in which the payoff order is $T > R > P > S$, in the Chicken game, the fact that being exploited by a defector is better than getting punished for mutual defection demands a payoff order $T > R > S > P$. If x under this payoff order denotes the fraction of cooperators in a population of actors, then the replicator dynamics (3) leads to

$$\frac{\mathrm{d}x}{\mathrm{d}t} = x(\pi_{\mathrm{C}} - \overline{\pi}) = x(1-x)(\pi_{\mathrm{C}} - \pi_{\mathrm{D}}), \qquad [10]$$

where the expected utility of a randomly chosen actor is $\overline{\pi} = x\pi_{\mathrm{C}} + (1-x)\pi_{\mathrm{D}}$, while the expected utility of cooperators is $\pi_{\mathrm{C}} = xR + (1-x)S$, and the expected utility of defectors is $\pi_{\mathrm{D}} = xT + (1-x)P$. The equality of expected utilities $\pi_{\mathrm{C}} = \pi_{\mathrm{D}}$ yields a dimorphic equilibrium

$$0 < x^* = \frac{S-P}{(T-R) + (S-P)} < 1. \qquad [11]$$

Rewriting the game payoffs in the spirit of the Donor-Recipient game in Eq. [2], we generally have $S = \Delta_1 > 0$, $T = R + \Delta_2$ ($\Delta_2 > 0$), and $P = 0$. In the matrix form

$$\mathbf{M}_{\mathrm{Ch}} = \begin{pmatrix} R & \Delta_1 \\ R + \Delta_2 & 0 \end{pmatrix}. \qquad [12]$$

The dimorphic equilibrium becomes

$$x^* = \frac{\Delta_1}{\Delta_1 + \Delta_2}. \qquad [13]$$

From the definition of the dilemma strength parameters

$$D'_{\mathrm{g}} \equiv \frac{T-R}{R-P} = \frac{\Delta_2}{R}, \qquad [14a]$$

$$D'_{\mathrm{r}} \equiv \frac{P-S}{R-P} = -\frac{\Delta_1}{R}. \qquad [14b]$$

Calculating the SED is a bit more convoluted than in the case of the Donor-Recipient game. First, we note that the equilibrium of mutual defection is always unstable because as $x \to 0^+$, then $\pi_\mathrm{C} \approx \Delta_1 > \pi_\mathrm{D} \approx 0$. Similarly, the equilibrium of mutual cooperation is always unstable because as $x \to 1^-$, then $\pi_\mathrm{C} \approx R < \pi_\mathrm{D} \approx R + \Delta_2$. The dimorphic equilibrium x^* is always stable. Because the expected utility can be rewritten as $\overline{\pi} = \overline{\pi}(x) = -x^2(\Delta_1 + \Delta_2) + x(R + \Delta_1 + \Delta_2)$, we obtain that

$$\Pi_\mathrm{NE} = \overline{\pi}(x^*) = \frac{\Delta_1}{\Delta_1 + \Delta_2}(R + \Delta_2) = x^*(R + \Delta_2). \qquad [15]$$

Furthermore, based on $\frac{\mathrm{d}\overline{\pi}}{\mathrm{d}x}\big|_{x=x_\mathrm{m}} = -2x_\mathrm{m}(\Delta_1 + \Delta_2) + R + \Delta_1 + \Delta_2 = 0$, the expected utility has a maximum at

$$x_\mathrm{m} = \frac{1}{2}\frac{R + \Delta_1 + \Delta_2}{\Delta_1 + \Delta_2} = \frac{1}{2}x^*\frac{R + \Delta_1 + \Delta_2}{\Delta_1}, \qquad [16]$$

which for $0 < R < \Delta_1 + \Delta_2$ falls between $\frac{1}{2} < x_\mathrm{m} < 1$. For $R \geq \Delta_1 + \Delta_2$, we have $x_\mathrm{m} \geq 1$, but because the fraction of cooperators cannot be greater than unity, the social optimum is achieved at $x_\mathrm{m} = 1$. Thus, the expected utility in the social optimum is

$$\Pi_\mathrm{SO} = \overline{\pi}(x_\mathrm{m}) = \begin{cases} \frac{1}{4}x^*\frac{(R+\Delta_1+\Delta_2)^2}{\Delta_1}, & 0 < R < \Delta_1 + \Delta_2 \\ R, & R \geq \Delta_1 + \Delta_2 \end{cases}. \qquad [17]$$

We finally obtain

$$\mathrm{SED} = \Pi_\mathrm{SO} - \Pi_\mathrm{NE} = \begin{cases} \frac{1}{4}x^*\frac{[R-(\Delta_1-\Delta_2)]^2}{\Delta_1}, & 0 < R < \Delta_1 + \Delta_2 \\ x^*\frac{\Delta_2}{\Delta_1}(R - \Delta_1), & R \geq \Delta_1 + \Delta_2 \end{cases}. \qquad [18]$$

If we additionally assume that $\Delta \equiv \Delta_1 = \Delta_2$ as in the main text, the result simplifies to

$$\mathrm{SED} = \Pi_\mathrm{SO} - \Pi_\mathrm{NE} = \begin{cases} \frac{R^2}{8\Delta}, & 0 < R < 2\Delta \\ \frac{1}{2}(R - \Delta), & R \geq 2\Delta \end{cases}. \qquad [19]$$

Supplementary Remark 4

The Public Goods Games are a multiplayer generalization of the Donor-Recipient game. This is best seen by starting from the unilateral payoff matrix for the latter game

$$\mathbf{U}_\text{D-R} = \begin{pmatrix} b & -c \\ 0 & 0 \end{pmatrix} \qquad [20]$$

which mathematically states that unilaterally cooperating confers a benefit b to another actor at a personal cost c, while unilaterally defecting does nothing. If we assumed that there were G actors involved, then choosing cooperation would incur a total personal cost $c' = Gc$. This cost can be thought of as putting the same amount into a common pool. With $j \leq G$ cooperators in the group, the pool contains the amount $jGc = jc'$. This amount is turned into the total benefit of $jGb = rjc'$, where $r \equiv \frac{b}{c}$ is a return factor. From these considerations, the payoff earned by a single cooperative actor in one game round is $r\frac{j}{G}c' - c'$, whereas the payoff earned by a single defecting actor is $r\frac{j}{G}c'$. The two expressions for the payoffs of cooperators and defectors have the same structure that arise from the common description of the Public Goods Games as outlined in the main text. This confirms that the Public Goods Games are indeed a multiplayer generalization of the Donor-Recipient game.

Suppose that in a large population, in which cooperators constitute a fraction x, actors consecutively form groups of size G to participate in an instance of the Public Goods Games. The expected utility of cooperators and defectors is then respectively given by

$$\pi_\mathrm{C}(x) = \sum_{j=0}^{j=G-1}\binom{G-1}{j}x^j(1-x)^{G-1-j}\left(r\frac{j+1}{G} - 1\right)c', \qquad [21a]$$

$$\pi_\mathrm{D}(x) = \sum_{j=0}^{j=G-1}\binom{G-1}{j}x^j(1-x)^{G-1-j}r\frac{j}{G}c'. \qquad [21b]$$

To interpret these equations, let us focus on the first one. The equation states that a cooperator happens to be in a group with j other cooperators with the binomial probability $\binom{G-1}{j}x^j(1-x)^{G-1-j}$, and then earns the payoff

$\left(r\frac{j}{G}-1\right)c'$. The sum runs across all possibilities, i.e., from $j=0$ to $j=G-1$ other cooperators. A similar reasoning applies to the second equation, with a distinction that here a defector happens to be in a group with j cooperators. Based on Eq. [10], the replicator dynamics is determined by the difference of the above-given utilities

$$\pi_{\mathrm{C}}(x)-\pi_{\mathrm{D}}(x)=\left(\frac{r}{G}-1\right)c'\sum_{j=0}^{j=G-1}\binom{G-1}{j}x^j(1-x)^{G-1-j}=\left(\frac{r}{G}-1\right)c'. \quad [22]$$

This identity shows that if $r<G$ (resp., $r>G$), then under the replicator dynamics in Eq. [10] defectors (resp., cooperators) prevail. The cost c' determines the speed of convergence. The expected utility is maximized when everyone cooperates, implying that $\Pi_{\mathrm{SO}}=\pi_{\mathrm{C}}(1)=(r-1)c'$. If $r>G$, then $\Pi_{\mathrm{NE}}=\Pi_{\mathrm{SO}}$ and $\mathrm{SED}=0$. Put alternatively, when the return factor r is large enough, the dilemma disappears. If, however, $r<G$, then $\Pi_{\mathrm{NE}}=\pi_{\mathrm{D}}(0)=0$ and

$$\mathrm{SED}=\Pi_{\mathrm{SO}}-\Pi_{\mathrm{NE}}=(r-1)c'-0=(r-1)c'. \quad [23]$$

Supplementary Remark 5

Public goods are a versatile concept that has proven useful beyond economics. A multitude of biological phenomena, especially in the microbial world (4), contain at their heart the same dilemma as the Public Goods Games. Cancer progression is one such phenomenon, in which cancer cells cooperate with one another via the secretion of diffusible factors (5). These diffusible factors can be considered public goods that benefit the tumor as a whole, but are under a constant threat by free-riding subclones.

One immediate difference between economic and biological public goods is that the benefit of receiving a diffusible factor, instead of being proportional to, saturates with increasing concentration (6). Mathematically, this corresponds to replacing the linear benefit $r\frac{j}{G}c'$ in Eqs. [21] with a saturating benefit function $b=b(j)$, e.g., the logistic function (6)

$$b(j)=\frac{1}{1+e^{s(k-j)}}, \quad [24]$$

where $0<k\leq G$ is an inflection point at which the benefit function has the steepness $s>0$. The difference in utilities in Eq. [22] now becomes

$$\pi_{\mathrm{C}}(x)-\pi_{\mathrm{D}}(x)=\sum_{j=0}^{j=G-1}\binom{G-1}{j}x^j(1-x)^{G-1-j}\left[b(j+1)-b(j)\right]-c'. \quad [25]$$

Intuition suggests that if we repeat an experiment $G-1$ times with a success probability of x, then the number of successes should be $j\approx(G-1)x$, or even $j\approx Gx$ as $G\to\infty$. This would further suggest that

$$\pi_{\mathrm{C}}(x)-\pi_{\mathrm{D}}(x)\approx\left[b(Gx+1)-b(Gx)\right]-c'. \quad [26]$$

Rewriting the benefit function as

$$b(Gx)\equiv b(x)=\frac{1}{1+e^{Gs(h-x)}}, \quad [27]$$

where $h\equiv\frac{k}{G}$, $0<h\leq 1$, is a fractional inflection point, yields

$$b(Gx+1)-b(Gx)=\frac{1}{G}\frac{b\left(x+\frac{1}{G}\right)-b(x)}{\frac{1}{G}}\xrightarrow[G\to\infty]{}\frac{1}{G}\frac{\mathrm{d}}{\mathrm{d}x}b(x), \quad [28]$$

and finally

$$\pi_{\mathrm{C}}(x)-\pi_{\mathrm{D}}(x)\approx\frac{1}{G}\frac{\mathrm{d}}{\mathrm{d}x}b(x)-c'. \quad [29]$$

The last equation allows for up to two internal equlibria $x^*_{1,2}$ on the domain $0<x<1$

$$x^*_{1,2}=h\mp\frac{2}{Gs}\mathrm{sech}^{-1}\left(2\sqrt{\frac{c}{s}}\right) \quad [30]$$

under the condition $0<c<\frac{s}{4}$. It can be shown that, as a consequence, one of five different types of dynamics arises (6):

- Type A dynamics occurs when $x = 0$ is the stable equilibrium, $x = 1$ is the unstable equilibrium, and there are no internal equilibria.
- Type B dynamics occurs when $x = 0$ and $x = 1$ are unstable, and there is one stable internal equilibrium $0 < x_2^* < 1$.
- Type C dynamics occurs when $x = 0$ is stable, $x = 1$ is unstable, and there are two internal equilibria $0 < x_1^* < x_2^* < 1$ among whom x_1^* is unstable and x_2^* is stable.
- Type D dynamics occurs when $x = 0$ and $x = 1$ are stable, and there is one unstable internal equilibrium $0 < x_1^* < 1$.
- Type E dynamics occurs when $x = 0$ is unstable, $x = 1$ is stable, and there are no internal equilibria.

The outlined analytical approximation for the difference of expected utilities given in Eq. [29] is useful to qualitatively understand the types of evolutionary dynamics that arise in Public Goods Games with the saturating benefit function $b = b(x)$. In simulations, however, we used the exact expression given in Eq. [25], but only after normalizing

$$b_{\mathrm{N}}(j) = \frac{b(j) - b(0)}{b(G) - b(0)}. \quad [31]$$

We examined the effects of three key parameters on the cooperation frequency and the SED. The first of the three parameters was the cost of producing diffusible factors c'. The second parameter was the diffusion range G, i.e., the number of cells that benefit when a diffusible factor is produced in their vicinity. Finally, the third parameter was the fractional inflection point h; with the steepness s fixed to $s = 0.5$, the parameter h roughly determines the proportion of producers within the diffusion range that is necessary for diffusible factors to confer a meaningful benefit to cells.

It is crucial to keep in mind that the current example is about cooperation among cancer cells, and that our goal is to undermine such cooperation. The results of numerical simulations reveal that increasing the values of parameters c' and G is detrimental for cooperation frequency (Fig. S1A). This is unsurprising for the following reasons. First, the benefit of receiving diffusible factors from producer cells is capped, which implies that there must exist a large enough cost of production that eclipses the benefit altogether. Second, sharing a fixed amount of diffusible factors among more non-producer cells dilutes the benefit. What turns out to be surprising, however, is that the cooperation frequency in the $G - c'$ plane remains nearly unfazed by an increase in the fractional inflection point from a small value of $h = 0.3$ to a large value of $h = 0.7$ (Fig. S1B). The only qualitative difference is that the B-type dynamics in the former case gives way to the D-type dynamics in the latter case. These last results are somewhat counterintuitive because we would expect that higher obstacles to conferring benefits via diffusible factors (i.e., a larger h) have a negative effect on cooperation.

When viewed through the prism of the SED, small-h and large-h situations are patently different (Fig. S1C, D). Specifically, in the part of the $G - c'$ plane where the C-type dynamics materializes, a small fractional inflection point makes cooperation within the cell system more efficient than a large fractional inflection point, as evidenced by the considerably different values of the SED in these two situations (Fig. S1C, D). When $h = 0.3$, the C-type domain is almost as efficient as the B-type domain, indicating that for most initial conditions (i.e., the initial densities of producer cells), the cell system ends in a largely cooperative state in which the production of diffusible factors is only slightly deficient compared to the optimum. When $h = 0.7$, by contrast, the C-type domain is almost as inefficient as the A-type domain, indicating that for most initial conditions, the cell system ends in a largely uncooperative state in which the production of diffusible factors is highly deficient compared to the optimum. Overall then, does the SED suggest that increasing the value of the parameter h is an actionable intervention target for disrupting cooperation among cancer cells?

The question of whether the parameter h, which measures the difficulty of conferring benefits via diffusible factors, is an actionable target for disrupting cancer-cell cooperation is an important one in the sense that drugs such as bevacizumab attempt to treat cancer by inhibiting diffusible factors, e.g., the vascular endothelial growth factor A (VEGF-A) (7). The SED-based analysis, however, gives little reason for optimism. In much of the phase space where producer cells resist free riding, and thus cancer as a whole maintains the cell heterogeneity necessary to metastasize, increasing the value of the parameter h has a very limited effect on the efficiency of the cancer-cell system as measured by the SED (Fig. S1E). Even when the system becomes considerably more inefficient, it still lands in C-type or D-type domains in which, depending on the initial conditions, producer cells may persist (Fig. S1E, F). Lastly, the gradient of the SED as an indicator of direction for incremental interventions never points solely towards increasing the value of the parameter h. In the h–c' plane, for example, the SED gradient invariably points in the direction of the increasing c' values, whereas simultaneously increasing the h values may or may not be helpful (Fig. S1E, F). The SED-based analysis thus suggests that on purely evolutionary grounds, the high hopes for bevacizumab-like drugs have been misplaced (8–10). Also of note is that, according to our results, bevacizumab-like drugs may be of help only in combination with treatments that target producer cells specifically (thus increasing the parameter c'). This,

unfortunately, is in sharp contrast with traditional chemotherapies that target cancer cells indiscriminately. A major synergy between bevacizumab and traditional chemotherapies is therefore unlikely.

Supplementary Remark 6

To explore the usefulness of the SED in a vaccination dilemma, we relied on an existing setup (11) that assumes an infinite well-mixed population in which the evolution of decision making is coupled with a periodic outbreak of a seasonal flu-like disease. A vaccination campaign is assumed to precede the flu season. Once the flu season starts, spreading is governed by a susceptible-infectious-recovered (SIR) process. Thereafter, actors compare how they fared during the epidemic against the performance of their peers, and probabilistically imitate one peer (12, 13). The probability of imitating the peer increases when the peer fared better than the actor. Conversely, the probability of imitating decreases when the actor fared better than the peer. Imitating a worse performer is improbable, but possible. The vaccine is assumed to be imperfect in that it fails to protect a fraction of vaccinated actors at random.

In the described setup, there are two dimensions of performance: (i) whether an actor vaccinated or chose to free ride, and (ii) whether the actor stayed healthy or contracted the disease. Accordingly, there are four possible outcomes:

(1) vaccinated and healthy actors pay only the vaccination cost c_{v};
(2) vaccinated but infected actors pay the infection cost c_{i} on top of the vaccination cost c_{v};
(3) free-riding but healthy actors pay zero cost; and
(4) free-riding and infected actors pay only the infection cost c_{i}.

There is no loss of generality if the infection cost is set to $c_{\mathrm{i}} = 1$ and then the relative vaccination cost is defined as $c_{\mathrm{r}} \equiv \frac{c_{\mathrm{v}}}{c_{\mathrm{i}}}$, $0 \le c_{\mathrm{r}} < 1$. The resulting payoffs can thus be written $\pi_{(i)} \in \{-c_{\mathrm{r}}, -c_{\mathrm{r}}-1, 0, -1\}$, $i = 1, 2, 3, 4$.

Beside payoffs associated with each of the four outcomes, we also need the corresponding probabilities of occurrence. These can be calculated based on the fact that the final epidemic size (i.e., the fraction of recovered actors at the end of the SIR process), $R(x,\infty)$, is given by the probability of being without immunity, $1 - xe$, and the probability of contracting the disease conditional on being non-immune, $p_{\mathrm{I|n\text{-}i}} = 1 - \exp\left[-R_0 R(x,\infty)\right]$. Precisely, we have

$$R(x,\infty) = (1 - xe)\, p_{\mathrm{I|n\text{-}i}} = (1 - xe)\left[1 - e^{-R_0 R(x,\infty)}\right], \qquad [32]$$

where x is the fraction of vaccinators, $0 < e \le 1$ is the vaccine efficacy, and R_0 is the basic reproduction number (14). Upon solving this transcendental equation for $R(x,\infty)$, the occurrence probabilities $p_{(i)}$, $i = 1, 2, 3, 4$, become:

(1) $x\left[e + (1-e)\left(1 - p_{\mathrm{I|n\text{-}i}}\right)\right]$ for vaccinated and healthy actors;
(2) $x(1-e)\, p_{\mathrm{I|n\text{-}i}}$ for vaccinated but infected actors;
(3) $(1-x)\left(1 - p_{\mathrm{I|n\text{-}i}}\right)$ for free-riding but healthy actors; and
(4) $(1-x)\, p_{\mathrm{I|n\text{-}i}}$ for free-riding and infected actors.

In addition to the payoffs $\pi_{(i)}$ and the corresponding probabilities $p_{(i)}$, we need one last bit of information that mutually connects the four possible outcomes. Specifically, we define that actors who experienced outcome (i) during the past epidemic season, imitate their peers who experienced outcome (j) with the probability

$$p_{(i)\leftarrow(j)} = \frac{1}{1 + \exp\left(-\frac{\pi_{(j)} - \pi_{(i)}}{\kappa}\right)}, \qquad [33]$$

where κ is the irrationality of selection in the sense that the larger the value of this parameter, the lower is the influence of the payoff difference on decisions to imitate. The dynamic equation for the fraction of vaccinators (i.e., cooperators) is no longer given by the replicator equation, but rather by a mean-field equation of the form

$$\frac{\mathrm{d}x}{\mathrm{d}t} = \sum_{i=1}^{2}\sum_{j=3}^{4} p_{(i)} p_{(j)} \left(p_{(j)\leftarrow(i)} - p_{(i)\leftarrow(j)}\right), \qquad [34]$$

At last, we are in a position to define the SED. The expected utility of vaccinators (i.e., cooperators) is

$$\pi_{\mathrm{C}}(x) = \pi_{(1)} p_{(1)} + \pi_{(2)} p_{(2)}, \qquad [35]$$

and the expected utility of free riders (i.e., defectors) is

$$\pi_{\mathrm{D}}(x) = \pi_{(3)} p_{(3)} + \pi_{(4)} p_{(4)}. \qquad [36]$$

With the usual expression for the expected utility of a randomly chosen actor $\overline{\pi}(x) = x\pi_{\mathrm{C}}(x) + (1-x)\,\pi_{\mathrm{D}}(x)$, we have $\Pi_{\mathrm{SO}} = \max_x \overline{\pi}(x)$ in the social optimum and $\Pi_{\mathrm{NE}} = \overline{\pi}(x^*)$ in the evolutionary equilibrium x^* obtained from Eq. [34]. If, in addition, the system is bistable for given values of key parameters, here c_{R} and e, the equilibrium fraction of cooperators depends on the initial condition x_0, i.e., $x^* = x^*(x_0)$. In such a case we perform additional averaging across all possible initial values $0 < x_0 < 1$, i.e., $\Pi_{\mathrm{NE}} = \int_0^1 \overline{\pi}[x^*(x_0)]\,\mathrm{d}x_0$. The SED is finally given by

$$\mathrm{SED} = \Pi_{\mathrm{SO}} - \Pi_{\mathrm{NE}} = \max_{x\in[0,\,1]} \overline{\pi}(x) - \int_0^1 \overline{\pi}[x^*(x_0)]\,\mathrm{d}x_0. \qquad [37]$$

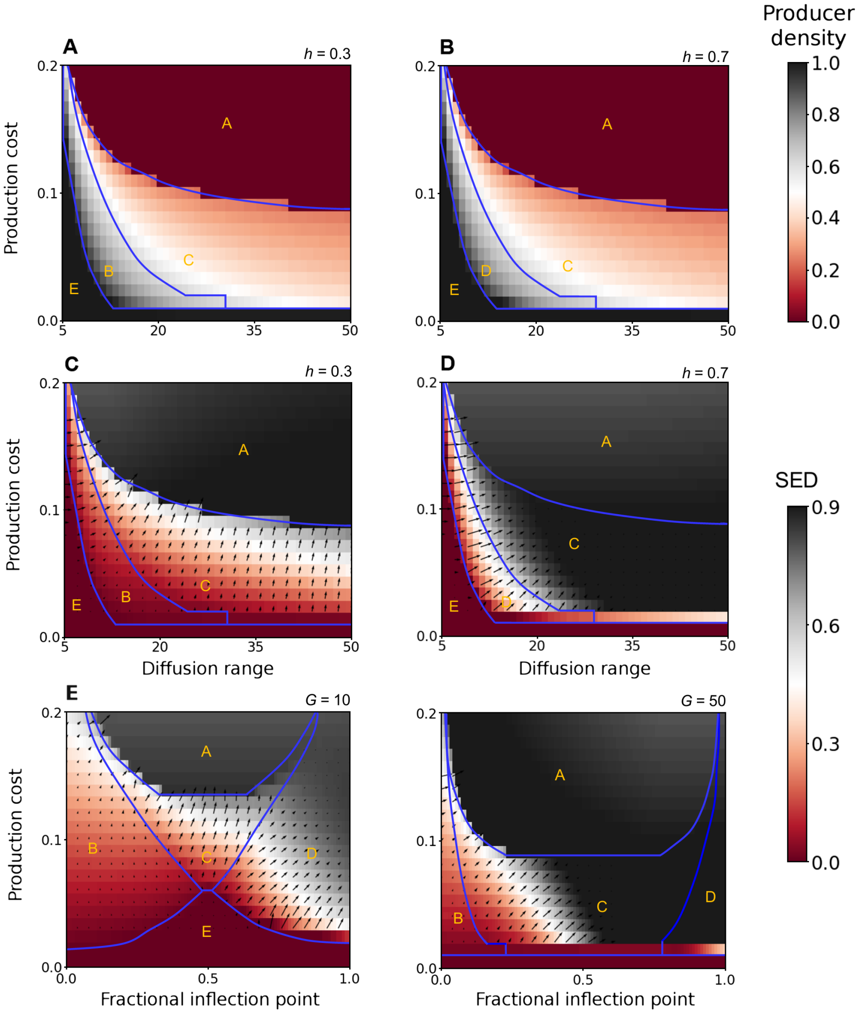

Fig. S1. Cancer metastasis is a cellulo-social phenomenon analyzable using the social efficiency deficit. Cancer cells benefit from the production of diffusible factors as a form of public good. However, such production is costly, making producer cells susceptible to free riding. **A, B,** Density of producer cells decreases with the increase of the production cost c' and the diffusion range G. Interestingly, changing the fractional inflection point h of the benefit function in Eq. [27] from a small value of $h = 0.3$ to a large value of $h = 0.7$ results in similar producer densities. This is unexpected because the parameter h quantifies the difficulty of conferring benefits via diffusible factors, and therefore should negatively affect cell cooperation. Note that the results in these plots are averaged over all possible initial conditions. Capital letters A – E denote the five types of dynamics defined in the text. **C, D,** Although the producer density stays the same irrespective of the h value, small-h and large-h situations are very different when examined using the social efficiency deficit. In particular, cancer cells under the C-type dynamics change from being fairly efficient to being rather inefficient in the sense of underproducing diffusible factors in the evolutionary equilibrium relative to the optimum. **E, F,** Bevacizumab-like drugs are designed to inhibit diffusible factors, which in the model corresponds to increasing the value of the parameter h. The social efficiency deficit, however, reveals that increasing the parameter h is unlikely to have more than a limited effect on cooperation among cancer cells; the cell system is indeed pushed towards the less efficient C-type or D-type dynamics, yet producer cells may still persist. The gradient field suggests that even when increasing the value of the parameter h is effective, this should be accompanied with simultaneous increases of the parameter c', which in medical terms would translate to therapies that target producer cells specifically.

References

1. C Taylor, MA Nowak, Transforming the dilemma. *Evolution* **61**, 2281–2292 (2007).
2. H Ohtsuki, MA Nowak, The replicator equation on graphs. *J. Theor. Biol.* **243**, 86–97 (2006).
3. J Hofbauer, K Sigmund, *Evolutionary games and population dynamics.* (Cambridge University Press), (1998).
4. B Allen, J Gore, MA Nowak, Spatial dilemmas of diffusible public goods. *Elife* **2**, e01169 (2013).
5. M Archetti, KJ Pienta, Cooperation among cancer cells: applying game theory to cancer. *Nat. Rev. Cancer* **19**, 110–117 (2019).
6. M Archetti, Evolutionary game theory of growth factor production: implications for tumour heterogeneity and resistance to therapies. *Br. J. Cancer* **109**, 1056–1062 (2013).
7. M Los, JM Roodhart, EE Voest, Target practice: lessons from phase III trials with bevacizumab and vatalanib in the treatment of advanced colorectal cancer. *Oncologist* **12**, 443 (2007).
8. DF Hayes, Bevacizumab treatment for solid tumors: boon or bust? *JAMA* **305**, 506–508 (2011).
9. V Ranpura, S Hapani, S Wu, Treatment-related mortality with bevacizumab in cancer patients: a meta-analysis. *JAMA* **305**, 487–494 (2011).
10. L Richards, Disappointing outcomes for anti-VEGF therapy. *Nat. Rev. Clin. Oncol.* **8**, S6–S6 (2011).
11. K Kuga, J Tanimoto, Which is more effective for suppressing an infectious disease: imperfect vaccination or defense against contagion? *J. Stat. Mech.: Theory Exp.* **2018**, 023407 (2018).
12. F Fu, DI Rosenbloom, L Wang, MA Nowak, Imitation dynamics of vaccination behaviour on social networks. *Proc. R. Soc. B* **278**, 42–49 (2010).
13. CT Bauch, Imitation dynamics predict vaccinating behaviour. *Proc. R. Soc. B* **272**, 1669–1675 (2005).
14. P Van den Driessche, J Watmough, Reproduction numbers and sub-threshold endemic equilibria for compartmental models of disease transmission. *Math. Biosci.* **180**, 29–48 (2002).
